# ABL1 kinase as a tumor suppressor in AML1-ETO and NUP98-PMX1 leukemias

**DOI:** 10.1038/s41408-023-00810-0

**Published:** 2023-03-23

**Authors:** Konstantin Golovine, Gleb Abalakov, Zhaorui Lian, Srinivas Chatla, Adam Karami, Kumaraswamy Naidu Chitrala, Jozef Madzo, Margaret Nieborowska-Skorska, Jian Huang, Tomasz Skorski

**Affiliations:** 1 Fels Cancer Institute for Personalized Medicine, Temple University Lewis Katz School of Medicine, Philadelphia, PA USA; 2 Coriell Institute for Medical Research, Camden, NJ USA

**Keywords:** Acute myeloid leukaemia, Cancer genetics

## Abstract

Deletion of *ABL1* was detected in a cohort of hematologic malignancies carrying AML1-ETO and NUP98 fusion proteins. *Abl1−/−* murine hematopoietic cells transduced with AML1-ETO and NUP98-PMX1 gained proliferation advantage when compared to *Abl1* + */+* counterparts. Conversely, overexpression and pharmacological stimulation of ABL1 kinase resulted in reduced proliferation. To pinpoint mechanisms facilitating the transformation of ABL1-deficient cells, *Abl1* was knocked down in 32Dcl3-*Abl1ko* cells by CRISPR/Cas9 followed by the challenge of growth factor withdrawal. 32Dcl3-*Abl1ko* cells but not 32Dcl3-*Abl1wt* cells generated growth factor-independent clones. RNA-seq implicated PI3K signaling as one of the dominant mechanisms contributing to growth factor independence in 32Dcl3-*Abl1ko* cells. PI3K inhibitor buparlisib exerted selective activity against Lin-cKit+ NUP98-PMX1;*Abl1−/−* cells when compared to the *Abl1* + */+* counterparts. Since the role of ABL1 in DNA damage response (DDR) is well established, we also tested the inhibitors of ATM (ATMi), ATR (ATRi) and DNA-PKcs (DNA-PKi). AML1-ETO;*Abl1−/−* and NUP98-PMX1;*Abl1−/−* cells were hypersensitive to DNA-PKi and ATRi, respectively, when compared to *Abl1* + */+* counterparts. Moreover, ABL1 kinase inhibitor enhanced the sensitivity to PI3K, DNA-PKcs and ATR inhibitors. In conclusion, we showed that ABL1 kinase plays a tumor suppressor role in hematological malignancies induced by AML1-ETO and NUP98-PMX1 and modulates the response to PI3K and/or DDR inhibitors.

## Introduction

The *ABL1* proto-oncogene encodes a cytoplasmic and nuclear protein tyrosine kinase, which has been implicated in regulation of differentiation, proliferation, adhesion, and stress response [1–3]. Cytoplasmic ABL1 stimulates proliferation whereas nuclear ABL1 negatively regulates cell growth [4, 5]. Although normal ABL1 kinase has no direct transforming activity, its tumor promoting function has been reported before [2]. For example, ABL1 was found to be overexpressed and activated in numerous solid tumors presumably to promote tumor cell growth and invasion. Constitutively activated oncogenic mutants of the ABL1 resulting from chromosomal translocations (BCR-ABL1, TEL-ABL1) or episomal amplification (NUP214-ABL1) induce acute and chronic leukemias [1].

Intriguingly, the tumor suppressor function of ABL1 kinase has also been suggested because its overexpression in fibroblasts induced cell cycle arrest in the G1 phase [3, 6–8]. This effect usually requires nuclear localization of the ABL1 protein and the presence of p53 and Rb [4, 9]. Conversely, loss of ABL1 facilitates transformation in the context of p53 and Rb deficiency [10] and in the presence of BCR-ABL1 [11].

Since our initial analysis detected *ABL1* deletions in leukemias carrying AML1-ETO and NUP98 translocations, we investigated the role of ABL1 in these leukemias. AML1-ETO is encoded by the t(8;21(q22;q22) translocation, which is one of the most frequent genetic alterations in acute myeloid leukemia (AML) [12]. Patients with AML-ETO-positive leukemias have a relatively favorable prognosis. However, substantial numbers of these patients eventually relapse. In addition, the outcome of older patients with AML1-ETO -positive AML who are not eligible for aggressive chemotherapy remains dismal. The presence of *NUP98* gene fusion defines a high-risk leukemia subset with unfavorable outcomes [13]. Therefore, a significant unmet medical need is apparent for AML patients carrying AML1-ETO and NUP98-fusions.

Here we show that normal ABL1 kinase plays a tumor suppressor role in hematological malignancies induced by AML1-ETO and NUP98-PMX1 and modulates the response to phosphatidylinositol-3 kinase (PI3K) and DNA damage response (DDR) inhibitors.

## Materials and Methods

### Mice breading and genotyping

*c-Abl1* + */+* and *c-Abl1−/−*, *c-Abl1* + */-* mice with conditional *Abl1* deletion in hematopoietic cells were generated by crossing *Vav-iCre* mice (Jackson Laboratory, stock # 008610) with *c-Abl1*^*f*^^*lox/flox*^, *c-Abl1*^*wt/flox*^, and *c-Abl1*^*wt/wt*^ mice, which were made by crossbreeding *c-Abl1*^*flox/wt*^ mice (Jackson Laboratory, stock # 024286). Mice were maintained at Temple University’s Health Science campus animal facilities following the guidelines of Institutional Animal Care and Use Committee (IACUC) of Temple University. Mice genotypes were confirmed by PCR using specific primers as recommended by Jackson Laboratory (www.jax.org) (Supplemental Table S1).

### CRISPR/Cas9 targeting

Briefly, guide RNAs (gRNAs) targeting murine *Abl1* were generated using CRISPR Design (Supplemental Table S2) and cloned into the lenti*CRISPRv2* vector. The lenti*CRISPRv2* vector with a gRNA insert, the packaging plasmid *psPAX2*, and the envelope plasmid VSVG were mixed together and packed in HEK293T cells using Fugene 6. Lentiviruses were harvested at 48 and 72 h, respectively. 32Dcl3 cells were infected with freshly collected *CRISPR-Cas9-gRNA* lentivirus supplemented with 8 μg/ml polybrene (Sigma-Aldrich) for 48 h. Infected cells were selected in media with puromycin (1 μg/ml) for up to 14 days and successful mutation of infected clones was confirmed with T7E1 assay. Single clones were selected. Downregulation of ABL1 protein was detected by Western blot. Clone 6 and 8 were used for the experiments.

### T7E1 assay

Genomic DNA was extracted from cells 72 hours after puromycin selection. PCR was performed with primers specific to g1 and g2 (Supplemental Table S2). Then T7E1 assay was carried out using the PCR products. Indels were detected in all 3 gRNA infected 32Dcl3 cells.

### Western blot

Total cell lysates were obtained as described before [14] and analyzed by SDS-PAGE using primary antibodies against ABL1 (Santa Cruz Biotechnology, sc-56887), phospho-ABL1 (Cell Signaling, 2865), AML1 (Cell Signaling, 4334), NUP98 (Santa Cruz Biotechnology, sc-101546), phospho-CHK1 (Cell Signaling, 2348), CHK1 (Cell Signaling 2360), phospho-CHK2 (Abcam, ab59408), CHK2 (Cell Signaling, 2662), phospho-S6K (Cell Signaling, 9204), S6K (Cell Signaling, 9202), phospho-AKT (Cell Signaling, 9271), AKT (Cell Signaling, 2920), γ-H2AX (Cell Signaling, 2577), histone H3 (Invitrogen, AHO1432) and β-actin (Santa Cruz Biotechnology, sc-47778). The following secondary antibodies conjugated to HRP were used: goat anti-mouse IgG (EMD Millipore, 12-349) and goat anti-rabbit IgG (EMD Millipore, 12-348).

### RNA-seq

RNA-seq was performed by Illumina HiSeq 2000 platform. The raw RNA-Seq data from the *Abl1*/IL3 + , *Abl1ko*/IL3 + and *Abl1ko*/IL3- mouse samples were processed using the Subread algorithm [15]. Analysis of the RNA-seq data was performed using *Rsubread* package, a bioconductor software package for fast and best alignment and quantification of RNA sequencing reads [16]. RNA transcripts were mapped to mouse genome reference consortium build 38 (GRCm38) genome by building the genome index using *buildindex* function followed by alignment using the *align* function in the *Rsubread* package. To summarize the data to integer-based, gene-level read counts we used the *featureCounts* function within the *Rsubread* package. Generated read counts were annotated using mm10 annotation. RNA-seq data are deposited in GSE214710.

### Pathway enrichment and gene functional analysis

Biochemical pathways and the gene ontology (GO) functions for the differentially expressed genes from RNA seq analysis was performed by using the DAVID database, a gene functional classification tool to perform functional analysis for analyze large gene lists [17]. DAVID provides the functional enrichment terms in the categories of biological process (BP), cellular component (CC), molecular function (MF) and Kyoto Encyclopedia of Genes and Genomes (KEGG) pathways. We have selected the genes that are altered at a threshold count of 2 and the Expression Analysis Systematic Explorer (EASE) count of 0.1 for our analysis.

### Plasmid constructions

*Nup98-Pmx1* gene was recloned from pcDNA3.1-Nup98-Pmx1 vector kindly gifted by Dr. T. Nakamura [T. Nakamura et.al., Blood, 1999, https://pubmed.ncbi.nlm.nih.gov/10397741/] into retroviral plasmid pCMMP-MCS-IRES-mRFP (Addgene, #36972) by XhoI and BamHI restriction sites. MigR1-AE9a plasmid containing *AML1-ETO-9a* gene was a gift from Dong-Er Zhang (Addgene plasmid # 12433). *AE9a* was also recloned into pMSCV-mCherryFP plasmid [18] by EcoRI and XhoI. To produce pLenti6.3-TO-V5-myrAbl-6xHis-Flag vector mouse myristoylated Abl1 gene was cut out from pcDNA3-Abl-His6-FLAG plasmid gifted from Benjamin Turk (Addgene plasmid # 52684) by KpnI blunted, and XbaI restriction and transferred into pLenti6.3-TO-V5-WT-ABL1-6xHis-Flag plasmid kindly provided by John Brognard [https://pubmed.ncbi.nlm.nih.gov/26758680/]. Nonmyristoilated *WT-ABL1* was cut out from pLenti6.3-TO-V5-WT-ABL1-6xHis-Flag by SpeI blunted and XbaI restrictions and replaced with mouse myristoylated *Abl1* gene.

### Retroviral and lentiviral infections

Ecotropic retroviruses were prepared by co-transfecting HEK 293 T/17 cells (ATCC® CRL-11268) in a 10-cm plate with 10 μg of packaging pCL-ECO (Addgene plasmid # 12371) and 10 μg retroviral based vectors by using Lipofectamine 2000 Transfection Reagent (Invirtogen) according to manufacturer’s protocol. For lentiviral infection, transfer vectors were co-transfected into HEK 293 T/17 cells with packaging pCMV delta R8.2 (Addgene plasmid # 12263) and envelope pVSV-G (Clontech, PT3343-5) vectors. Viruses were harvested 40 h and 64 h after the transfection and filtered through a 0.45-µm PES filter (Millipore). For viral infections, 5 × 10^5^ mouse bone marrow cells or 32Dcl3 cells were resuspended in 1 ml of virus-containing medium with 6 μg/ml polybrene (Sigma). GFP, RFP or mCherryFP -positive cells were sorted 48 h after the initial infection.

### Generation of tetracycline-inducible cell lines

Lentiviral infections were performed with the pLenti6.3-TO-V5-myrAbl-6xHis-Flag viral construct or pLenti3.3/TR vector (for tetracycline repressor expression) using the ViraPower HiPerform T-Rex Gateway Expression System (Invitrogen) following the manufacturer’s instructions. 32Dcl3 cells were infected first with TR lentiviral stock and then with the myristoylated *ABL1* lentiviral stocks on two consecutive days in the presence of 6 µg/ml polybrene (Sigma). Cells expressing both constructs were selected for over 10 days using 10 μg/ml blasticidin (Invitrogen) and 500 µg/ml geneticin (Gibco). *NUP98-PMX1* or *AML1-ETO-9a* cDNAs were introduced into Tet-inducible Abl1-32Dcl3 cells by retroviral infections. A total of 1 µg/ml of tetracycline (Invitrogen) was used to induce expression of myristoylated wild-type mouse Abl1 in 32Dcl3 cells.

### Cell proliferation assays

Viable cells were counted using Trypan blue exclusion test. Cells were also plated in MethoCult H4230 (StemCell Technologies) and colonies were counted after 7–10 days.

### Inhibitors and drugs

Buparlisib (BKM120, #S2247), olaparib (AZD2281, #S1060), doxorubicin (Adriamycin, #S1208), NU7026 (#S2893), KU-60019 (#S1570), imatinib (STI571, #S1026) were from Selleck Chemicals. Rapamycin (sirolimus, #GC15031) was from GlpBio Technology. 6-hydroxy-DL-dopa (#H2380), ATR inhibitor IV (#504972), 5-(1,3-diaryl-1H-pyrazol-4-yl)hydantoin (DPH) (#SML0202) and tetracycline hydrochloride (#T3383) were from Millipore Sigma.

### Statistical analysis

Data are expressed as mean ± standard deviation (SD) from at least 3 independent experiments unless stated otherwise. When conducting subgroup comparisons between two groups, two-tailed unpaired t-test was used for normally distributed variables. p values less than 0.05 were considered statistically significant.

## Results

### *Abl1* knockout facilitated malignant phenotype of AML1-ETO and NUP98-PMX1 –positive cells

**Fig. 1:**
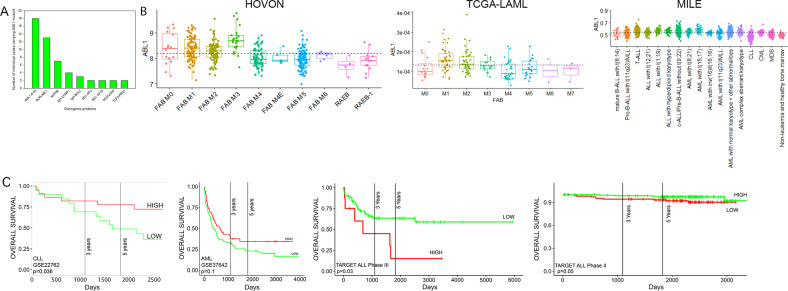
ABL1 as a prognostic factor. **A** Chromosomal aberrations detected in leukemias carrying del(9q34) or deletions encompassing this region resulting in loss of *ABL1* in CGAP Mitelman database (https://mitelmandatabase.isb-cgc.org/). **B** Normalized *ABL1* expression from HOVON, TCGA-LAML, and MILE (left to right) datasets. HOVON and MILE datasets were sourced from Affymetrix microarrays and normalized using the RMA method, while the TCGA-LAML dataset is is a set of rlog-normalized RNA-seq expression values. **C** Kaplan-Meier estimates of the overall survival of patients with indicated leukemias based on *ABL1* expression levels. HIGH and LOW cohorts in AML GSE22762 were divided at mean of the gene expression. Cutoffs for the AML dataset GSE37642 and the ALL datasets from TARGET ALL phases II and III (9.81, 4.05, and 10.88 of the RMA-normalized Affymetrix *ABL1* expression values, respectively) were determined using the maxstat package in R, an extension of the Hothorn and Lausen (2002) method to select maximally selected cut points. *p* values were calculated from the log-rank test.

The analysis of CGAP Mitelman database [see Supplementary Table 1 in [11]] showed that ~94% of malignancies carrying del(9q34) were of hematopoietic origin, suggesting that loss of *ABL1* may alter malignant transformation preferentially in the hematopoietic cells. Most of the cases carried complex karyotypes, but we identified the following chromosomal translocations in del(9q34) cases (Fig. 1A): t(8;21) AML1-ETO [t(8;21) in 14% of hematologic cases], BCR-ABL1 [t(9;22) in 10%], NUP98 translocations [t(1;11), t(2;11) and t(7;11) in 5%], CCND1 translocations [t(11;14) in 3%], BCL2 translocations [t(14;18) in 2%), MLL translocations [t(4;11) MLL-AF4 and t(10;11) MLL-AF10 in 3%], MOZ-p300 [t(8;22)] and TCF3-PBX1 [t(1;19)] (each in 1.5%). These data implicate ABL1 loss in leukemias carrying chromosomal translocations other than t(9;22) encoding for BCR-ABL1 [11]. In addition, RNA-seq analyses revealed broad spectrum of *ABL1* expression in various leukemias (Fig. 1B). Overall survival of patients with leukemias in the context of *ABL1* expression levels revealed that chronic lymphocytic leukemia (CLL) and AML displaying low levels of *ABL1* mRNA expression had shorter overall survival, while opposite effect was detected in ALL (Fig. 1C). In addition, low *ABL1* expression had a negative impact on survival of patients with small cell lung carcinoma, ovarian carcinoma, and breast carcinoma (Supplemental Fig. S1). Altogether, *ABL1* expression level could be considered a prognostic factor in selected hematological malignancies and solid tumors.

The top genetic aberrations in hematological malignancies, other that BCR-ABL1 were t(8;21) generating fusion protein AML1-ETO and NUP98 translocations, e.g., t(1;11) encoding NUP98-PMX1. To test the role of ABL1 in leukemogenesis mediated by these genetic aberrations *Abl1−/−* and *Abl1* + */+* murine hematopoietic cells were employed. Since homozygous disruption of *Abl1* in mice caused neonatal lethality/poor viability [19, 20], first we generated *Abl1−/−;Vav-Cre* (*n* = 19), *Abl1* + */-;Vav-Cre* (*n* = 52) and *Abl1* + */+;Vav-Cre* (*n* = 13) mice. Vav-Cre starts to express specifically in hematopoiesis system after the birth and can knock out *Abl1* efficiently, which has been confirmed by PCR using peripheral blood leukocytes from the peripheral blood samples and tail tissues (Supplemental Fig. S2A, B). These mice served as bone marrow donors to study leukemic transformation.

**Fig. 2:**
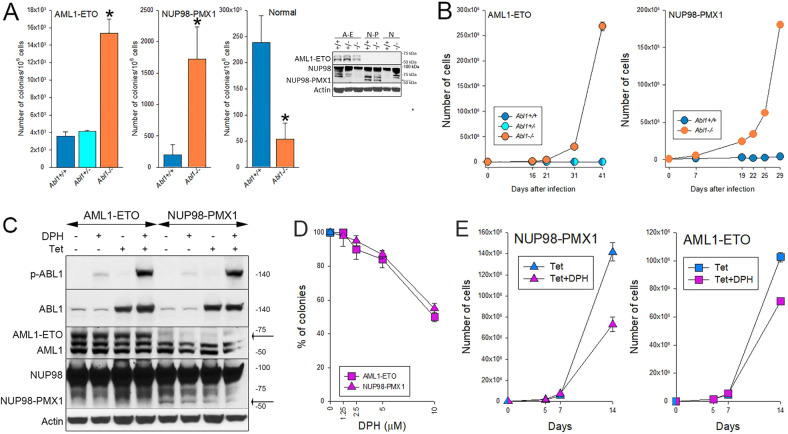
ABL1 regulated proliferation of murine hematopoietic cells expressing AML1-ETO and NUP98-PMX1. **A**, **B** Murine bone marrow cells from *Abl1−/−*, *Abl* + */-* and *Abl1* + */+* mice (3 mice/group) were infected with retroviruses and lentiviruses carrying *AML1-ETO* and *mCherry*, *NUP98-PMX1* and *GFP*, and *mCherry* or *GFP* only. **A** Clonogenic activity; Insect: Western blot detecting indicated proteins using anti-AML1 and anti-NUP98 antibodies (arrow indicates the position of NUP98-PMX1 protein). Results represent mean + /- SD from triplicate experiments; p < 0.001 using Student t-test. **B** total number of mCherry+ and GFP + cells in liquid culture. **C**–**E** AML1-ETO and NUP98-PMX1 -positive tet-inducible ABL1-32Dcl3 cells were maintained in Tet+ or Tet- medium in the presence or absence of DPH. **C** Western analysis of phospho-Y412 ABL1 (p-ABL1), total ABL1, AML1-ETO, NUP98-PMX1 and actin in the indicated cells incubated or not with 10 μM DPH for 30 minutes (arrows indicate the position of AML1-ETO and NUP98-PMX1 proteins). **D** Clonogenic activity of AML1-ETO and NUP98-PMX1 -positive cells maintained in Tet+ medium and incubated in the presence of indicated concentrations of DPH for 4 days followed by plating in MethoCult. Results represent % of colonies ± SD when compared to DPH-untreated cells. **E** proliferation of AML1-ETO and NUP98-PMX1 -positive cells maintained in Tet+ medium in the presence or absence of 10 μM DPH. Results represent number of living cells detected by trypan blue exclusion ± SD.

The results clearly show that in the absence of *Abl1*, Lin-c-Kit+ AML1-ETO and NUP98-PMX1 cells displayed higher clonogenic activity (Fig. 2A) and gained tremendous proliferation advantage in long-term tissue culture (Fig. 2B). Conversely, the absence of *Abl1* reduced the proliferation potential of normal Lin-c-Kit+ hematopoietic cells. In addition, G-CSF-induced myeloid differentiation of AML1-ETO;*Abl1*^*−/−*^ cells was reduced when compared to AML1-ETO;*Abl* + */+*^*-*^ cells (Supplemental Fig. S2C). However, myeloid differentiation of NUP98-PMX1;*Abl1*^*−/−*^ cells and non-transformed *Abl1−/−* was not significantly different from that in *Abl1* + */+* counterparts (Supplemental Fig. S2D, E). This inconsistency in the impact of ABL1 on differentiation might depend on the differences between AML1-ETO and NUP98-PMX1 in regulating differentiation by downregulation of C/EBPα and c-FOS, respectively [21, 22]. This speculation is supported by the observations that C/EBPα was downregulated in BCR-ABL1 -positive cells in which loss of ABL1 attenuated differentiation [11, 23].

To further explore the effect of activation of ABL1 kinase on proliferation of leukemia cells, tet-inducible ABL1-32Dcl3 cells were transduced with AML1-ETO and NUP98-PMX1 (Fig. 2C). ABL1 was overexpressed upon the treatment with tetracycline and ABL1 kinase was stimulated by DPH, an agonist of ABL1 kinase [24] (Fig. 2C). DPH-mediated activation of overexpressed ABL1 kinase was associated with reduced clonogenic activity (Fig. 2D) and proliferation (Fig. 2E) of 32Dcl3 cells.

### ABL1 regulated the sensitivity of NUP98-PMX1 murine leukemia cells to the intracellular signaling inhibitors

**Fig. 3:**
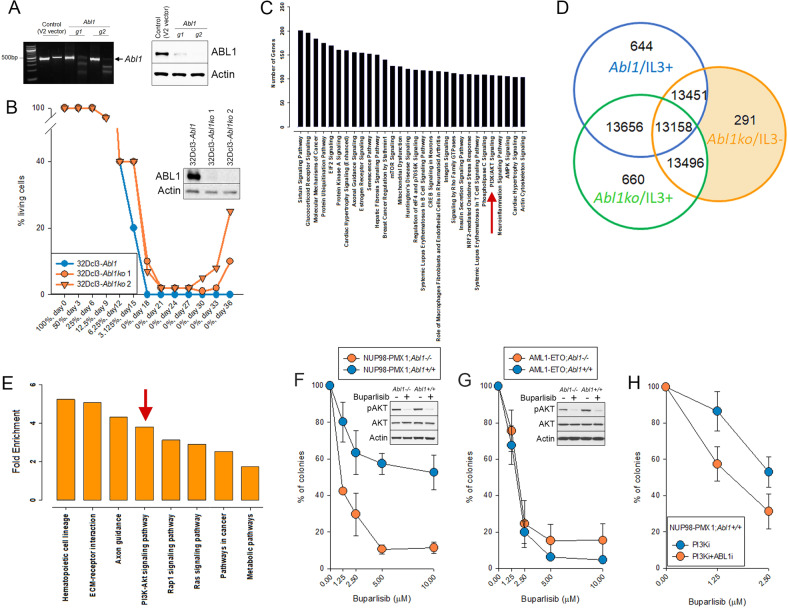
The impact of ABL1 on response to PI3K inhibitor. **A** CRISPR/Cas9-mediated deletion of *Abl1* in 32Dcl3-*Abl1ko* cells. Left panel – *Abl1* detected by T7E1 assay. Right panel – Western blot of ABL1 and Actin. **B** Cell survival curves of 32Dcl3-*Abl1* (Control V2 vector) and 32Dcl3-*Abl1ko* cells following gradual IL-3 withdrawal. Inset - Expression of ABL1 and Actin in two 32Dcl3-*Abl1ko* clones (1 and 2) growing without IL-3 and in 32Dcl3-*Abl1* cells growing in the presence of IL-3 for 2 months after the start of the experiment. **C** Ingenuity Pathway Analysis (IPA) of the genes deregulated in the 32Dcl3-*Abl1ko/IL3-* cells using Fragments per Kilobase per Million mapped reads (FPKM) values. Analysis demonstrating top 30 significant canonical pathways. **D** Venn diagram showing the number of genes which expression is uniquely deregulated in 32Dcl3-*Abl1ko* cells growing in the absence of IL-3 when compared to 32Dcl3-*Abl1ko* and 32Dcl3-*Abl1* cells growing in the presence of IL-3. **E** KEGG (Kyoto Encyclopedia of Genes and Genomes) pathways that are altered in the genes at a threshold count of 2 and EASE (the Expression Analysis Systematic Explorer) value of 0.1. Histogram bars represent the fold enrichment score calculated for genes in each pathway. PI3K/AKT pathway is marked by red arrow in panels **C** and **E**. **F, G** Lin-c-Kit+ NUP98-PMX1;*Abl1−/−*, NUP98-PMX1;*Abl1* + */+*, AML1-ETO;*Abl1−/−*, and AML1-ETO;*Abl1* + */+* murine bone marrow cells were treated with the indicated concentrations of PI3K inhibitor buparlisib. After 72 h cells were plated in methylcellulose and colonies were counted 7 days later. Results represent mean % ± SD of colonies when compared to untreated controls. Insets: cells were treated with 5 μM buparlisib for 24 h. Total cell lysates were analyzed by Western blot detecting indicated proteins (p = phosphorylated). **H** Lin-c-Kit+ NUP98-PMX1;*Abl1* + */+* cells were treated or not with 2μM ABL1i imatinib + /− the indicated concentrations of buparlisib. After 72 h cells were plated in methylcellulose and colonies were counted 7 days later. Results represent mean % ± SD of colonies when compared to untreated controls.

Growth factor independence is an important step in malignant transformation of hematopoietic cells [25]. To pinpoint mechanisms collaborating with *Abl1* deletion in transformation of hematopoietic cells, *Abl1* was knocked down in 32Dcl3 cells by CRISPR/Cas9 (using two gRNAs, g1 and g2, see Supplemental Table S2) to obtain 32Dcl3-*Abl1ko* cells (Fig. 3A). 32Dcl3-*Abl1* (Control, V2 vector) and 32Dcl3-*Abl1ko* cells were challenged to achieve early transformation potential by starving them from growth factors (IL3). Only 32Dcl3-*Abl1ko* cell populations (1 and 2, derived from g1 and g2, respectively) generated growth factor-independent cells (Fig. 3B). This indicates that the absence of *Abl1* may facilitate malignant transformation of hematopoietic cells.

RNA-seq was performed to compare gene expression in 32Dcl3-*Abl1* and 32Dcl3-*Abl1ko* cells in the presence or absence of IL-3. Pathway analysis of FPKM reads in IL3-independent (32Dcl3-*Abl1ko/IL3-*) cells showed altered expression of major genes involved in different biological pathways (Supplemental Table S3). Results from the pathway analysis showed that the elements of PI3K-AKT signaling pathway are enriched among the top 30 significant canonical pathways in 32Dcl3-*Abl1ko/*IL3- cells (Fig. 3C).

Furthermore, to identify the genes and pathways that are uniquely expressed in 32Dcl3-*Abl1ko/*IL3- cells compared to 32Dcl3-*Abl1*/IL3 + and 32Dcl3-*Abl1ko*/IL3 + cells, we performed Venn diagram analysis (Fig. 3D) followed by KEGG pathway analysis (Fig. 3E). RNA-seq analysis detected 291 genes uniquely expressed in 32Dcl3-*Abl1ko*/IL-3- cells when compared to 32Dcl3-*Abl1*/IL-3+ cells and 32Dcl3-*Abl1ko*/IL-3+ cells (Fig. 3D, Supplemental Table S4). Again, PI3K-AKT signaling pathway was significantly altered in 32Dcl3-*Abl1ko*/IL-3- cells (Fig. 3E).

Since PI3K-AKT pathway was identified in two independent analyses, we tested the sensitivity of AML1-ETO and NUP98-PMX1 –positive *Abl1−/−* and *Abl1* + */+* cells to PI3K inhibitor buparlisib (NVP-BKM120), a selective inhibitor of PI3K p110α/β/δ/γ subunits [26] which reduced phosphorylation of AKT (Fig. 3F, G**left panels**, insets). NUP98-PMX1;*Abl1−/−* murine leukemia cells were highly sensitive to buparlisib when compared to NUP98-PMX1;*Abl1* + */+* counterparts (Fig. 3F**, left panel**), at the same time ABL1 did not affect the sensitivity of AML1-ETO cells to the inhibitor (Fig. 3G**, left panel**).

To test if ABL1-positive NUP98-PMX1 leukemia cells may be sensitized to PI3K inhibitor, cells were treated with ABL1 kinase inhibitor imatinib and PI3K inhibitor buparlisib followed by clonogenic assay. The results clearly show that imatinib increased the sensitivity of NUP98-PMX1 leukemia cells to PI3Ki (Fig. 3H).

FPKM and KEGG pathway analyzes suggested potential involvement of mechanisms interacting with PI3K-AKT, including mTOR and RAS-RAF1 [27–29] (Fig. 3C–E). mTOR inhibitor rapamycin [30] inhibited phosphorylation of S6 kinase (Supplemental Fig. S3A, B, insets) and was selectively toxic for NUP98-PMX1;*Abl1−/−* cells when compared to NUP98-PMX1;*Abl1* + */+* counterparts (Supplemental Fig. S3A), whereas AML1-ETO -positive *Abl1−/−* and *Abl1* + */+* cells were equally sensitive to the inhibitor (Supplemental Fig. S3B). The presence or absence of ABL1 did not affect the sensitivity of AML1-ETO and NUP98-PMX1 –positive cells to RAF1 inhibitor LY3009120 (data not shown).

In summary, NUP98-PMX1;*Abl1−/−* cells were highly sensitive to PI3K and mTOR inhibitors when compared to the *Abl1* + */+* counterparts, and imatinib sensitized *Abl1* + */+* leukemia cells to PI3K inhibitor buparlisib. The resistance of NUP98-PMX1;*Abl1* + */+* cells PI3K and mTOR inhibitors might depend on the activation of a pathway redundant to PI3K/AKT/mTOR [31].

### ABL1 regulated the sensitivity of AML1-ETO and NUP98-PMX1 -positive cells to DDR inhibitors

Since the role of ABL1 in DDR is well established [32], we tested the sensitivity of AML1-ETO;*Abl1*^*−/−*^ and NUP98-PMX1;*Abl1*^*−/−*^ Lin-c-Kit+ cells to the inhibitors of DDR and also to DNA damaging agent doxorubicin to identify potential therapeutic vulnerabilities.

**Fig. 4:**
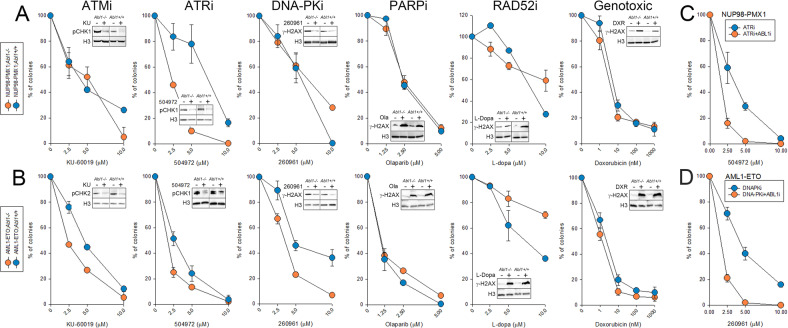
The impact of ABL1 on response to the drugs affecting DDR. **A** Lin-c-Kit+ NUP98-PMX1;*Abl1−/−* and NUP98-PMX1;*Abl1* + */+* cells, and **B** Lin-c-Kit+ AML1-ETO;*Abl1−/−* and AML1-ETO;*Abl1* + */+* cells were treated with the indicated concentrations of doxorubicin, PARPi olaparib, RAD52i 6-hydroxy-DL-dopa, ATMi KU-60019, ATRi 504972 and DNA-PKcsi 260961. After 72 h cells were plated in methylcellulose and colonies were counted 7 days later. Results represent mean % ± SD of colonies when compared to untreated controls (3 independent experiments). Insets: cells were treated with the inhibitors for 24 h. Total cell lysates were analyzed by Western blot detecting indicated proteins (p = phosphorylated). **C**, **D** Lin-c-Kit+ NUP98-PMX1;*Abl1* + */+* and AML1-ETO;*Abl1* + */+* cells were treated or not with 2 μM ABL1i imatinib + /- the indicated concentrations of DNA-PKcsi and ATRi. After 72 h cells were plated in methylcellulose and colonies were counted 7 days later. Results represent mean % ± SD of colonies when compared to untreated controls (3 independent experiments).

ABL1 kinase functionally interacts with DNA-PKcs, ATM and ATR, three major serine/threonine kinases regulating DDR [33, 34]. Therefore, we treated AML1-ETO;*Abl1*^*−/−*^ and NUP98-PMX1;*Abl1*^*−/−*^ cells and their *Abl1* + */+* counterparts with ATM inhibitor (ATMi) KU-60019, ATR inhibitor (ATRi) 504972 and DNA-PKcs inhibitor (DNA-PKi) 260961. Inhibition of these kinases was confirmed by Western blots detecting reduced phosphorylation of their substrates (CHK1, CHK2, H2AX) (Fig. 4A, B**, ATMi, ATRi, DNA-PKi** insets). AML1-ETO;*Abl1*^*−/−*^ cells were modestly more sensitive to all three inhibitors when compared to AML1-ETO;*Abl1* + */+* cells (Fig. 4A**, ATMi, ATRi, DNA-PKi**). Remarkably, NUP98-PMX1;*Abl1*^*−/−*^ cells were exceptionally sensitive to ATRi, but displayed similar sensitivity to ATMi and DNA-PKi when compared to NUP98-PMX1;*Abl*^*+/+*^ cells.

In addition, ABL1 kinase phosphorylates and regulates the activity of two DNA repair enzymes, PARP1 and RAD52, inhibition of which triggered synthetic lethality in BRCA1/2-deficient leukemia cells [14, 35–37]. We and others reported that AML1-ETO caused “BRCAness” phenotype in leukemia cells [35, 38], but loss of ABL1 expression did not sensitized leukemia cells to PARPi olaparib and RAD52i 6-hydroxy-DL-Dopa (Fig. 4A, B**, PARPi and RAD52i**). Both inhibitors were validated by detection of elevated levels of DNA double-strand breaks (DSBs marked by γ-H2AX) in the treated cells (Fig. 4A, B**, PARPi** and **RAD52i** insets).

Although ABL1 kinase affected survival/apoptosis after genotoxic treatment by regulation of p53 and p73 [39–42], it did not affect the sensitivity of AML1-ETO and NUP98-PMX1 -positive cells to doxorubicin (Fig. 4A, B**, Genotoxic**). Genotoxic effect of doxorubicin was confirmed by elevated levels of DSBs marked by γ-H2AX (Fig. 4A, B**, Genotoxic**, insets).

To test if ABL1-positive NUP98-PMX1 and AML1-ETO leukemia cells may be sensitized to ATRi and DNA-PKi, respectively, cells were treated with ABL1 kinase inhibitor imatinib and 504972 and 260961 compounds followed by clonogenic assay. The results clearly show that imatinib increased the sensitivity of NUP98-PMX1 and AML1-ETO leukemia cells to ATRi and DNA-PKi, respectively (Fig. 4C, D).

## Discussion

AML1-ETO and NUP98-fusions are usually accompanied by recurrent cooperating genetic events, for example *FLT3(ITD), WT1, KRAS, NRAS, KIT, MYC, NOTCH1, ASXL1, IDH1/IDH2* mutations, which contribute to leukemic transformation and/or accelerating more malignant disease progression [12, 13]. Here we reported that a loss of *ABL1* could be also detected in a cohort of leukemias carrying AML1-ETO and NUP98-fusions and that murine hematopoietic cells expressing AML1-ETO and NUP98-PMX1 displayed accelerated proliferation and/or reduced differentiation in the absence of *Abl1*. This observation is supported by our previous report that normal ABL1 kinase exerted tumor suppressor activity in leukemias expressing oncogenic forms of the kinase such as BCR-ABL1, TEL-ABL1 and NUP214-ABL1 [11]. The absence of ABL1 facilitated proliferation and genomic instability while abrogating differentiation of these leukemia cells resulting in highly malignant leukemia phenotypes. Altogether, we postulate that ABL1 is a tumor suppressor in myeloid malignancies.

Loss of *ABL1* has been previously reported as a rare but recurrent genetic abnormality in B- and T- ALL, but its impact on treatment response and prognosis is not known [43, 44]. We report here that low expression of *ABL1* might be a favorable prognostic factor in ALL.

ABL1 kinase can be also a therapeutic target. We reported that stimulation of the normal ABL1 kinase by a chemical compound DPH [24] enhanced anti-leukemia effect of TKIs such as imatinib and ponatinib in human and murine leukemias expressing BCR-ABL1, TEL-ABL1 and NUP214-ABL1 [11]. Here we show that ABL1 kinase inhibitor imatinib can be applied to increase the anti-leukemia effect of DNA damage response inhibitors (DNA-PKi and ATRi) and intracellular signaling inhibitors (PI3Ki) in AML1-ETO and/or NUP98-PMX1 leukemia cells. The effect most likely depended on inhibition of ABL1 kinase, but we cannot exclude potential off-target effects of imatinib [45].

In summary, *ABL1* loss may serve as important diagnostic factor predicting more malignant phenotype of AML1-ETO and NUP98-fusions -positive leukemias as well as their unique pharmaceutical vulnerabilities to PI3K, ATR and DNA-PK inhibition. On the other hand, ABL1 kinase could become a therapeutic target since imatinib increased the sensitivity of AML1-ETO and NUP98-fusions -positive leukemias to PI3K, ATR and DNA-PK inhibitors.

## Supplementary Information

Supplemental Material

## Data Availability

RNA-seq data are deposited in GSE214710.
