# Supplementary material for: ABL1 kinase as a tumor suppressor in AML1-ETO and NUP98-PMX1 leukemias
Source: Blood Cancer J. 2023 Mar 23;13(1):42. doi: 10.1038/s41408-023-00810-0 (PMC10036529; doi:10.1038/s41408-023-00810-0)
Supplement: Supplementary file 1 — Supplemental Material [file 41408_2023_810_MOESM1_ESM.pdf]

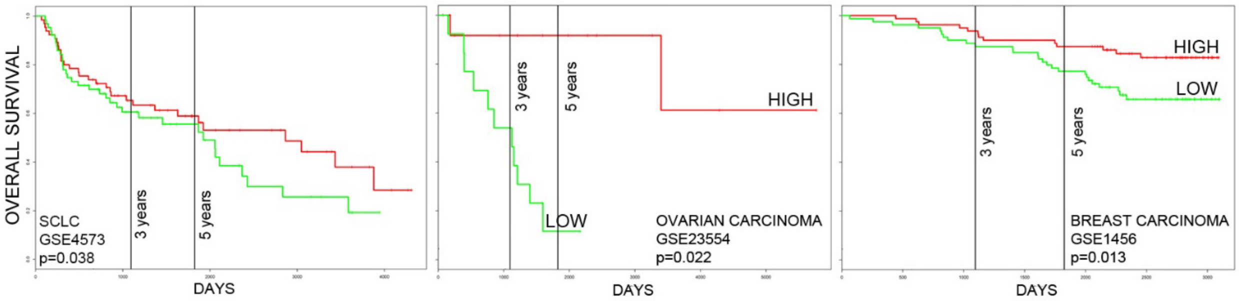

Supplemental Figure S1 (related to Figure 1). Kaplan-Meier estimates of the overall survival of patients with indicated solid tumors. HIGH and LOW cohorts were divided at mean of the gene expression; p values were calculated from the long rank test.

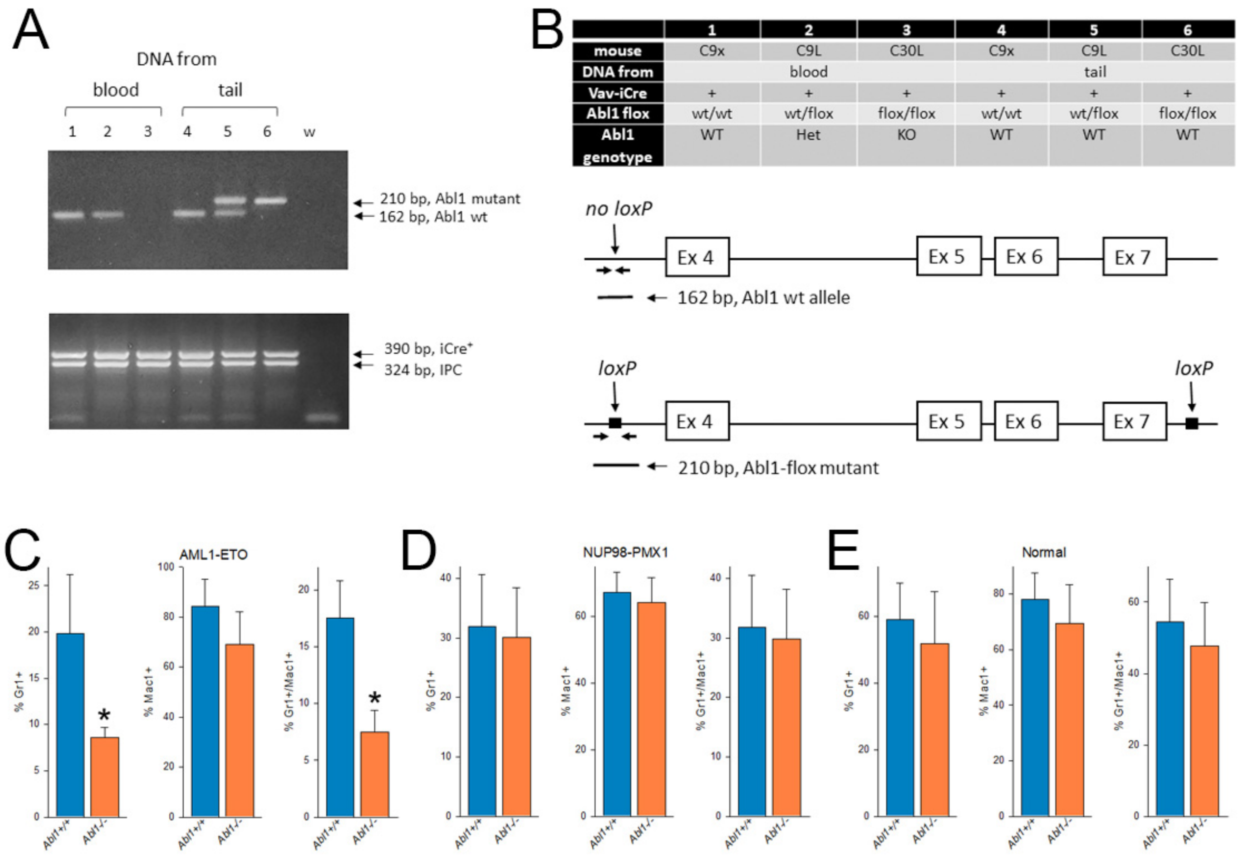

Supplemental Figure S2 (related to Figure 2). (**A**) Genotyping of *Abl1/Cre* mice using PCR analysis. DNA was isolated either from mouse tail or peripheral blood. IPC = internal positive control, w = water as negative control. *Abl1*-floxed allele and *Abl1* wild-type allele generated 210 bp band and 162 bp band, respectively. (**B**) *Abl1-flox* mice possess *loxP* site in the *Abl1* gene. Vav-iCre transgenic mice express Cre recombinase (iCre) in hematopoietic cells. Cre-mediated recombination results in deletion of the floxed sequence. Lin-c-Kit+ AML1-ETO (**D**), NUP-PMX1 and normal (**E**) cells were cultured in a threshold concentrations of growth factors + G-GSF followed by detection of Gr1+, Mac1+ and Gr1+Mac1+ cells. Results represent mean % +/- SD of Gr1+ and/or Mac1+ cells from triplicate experiments; $p<0.05$ using Student t-test.

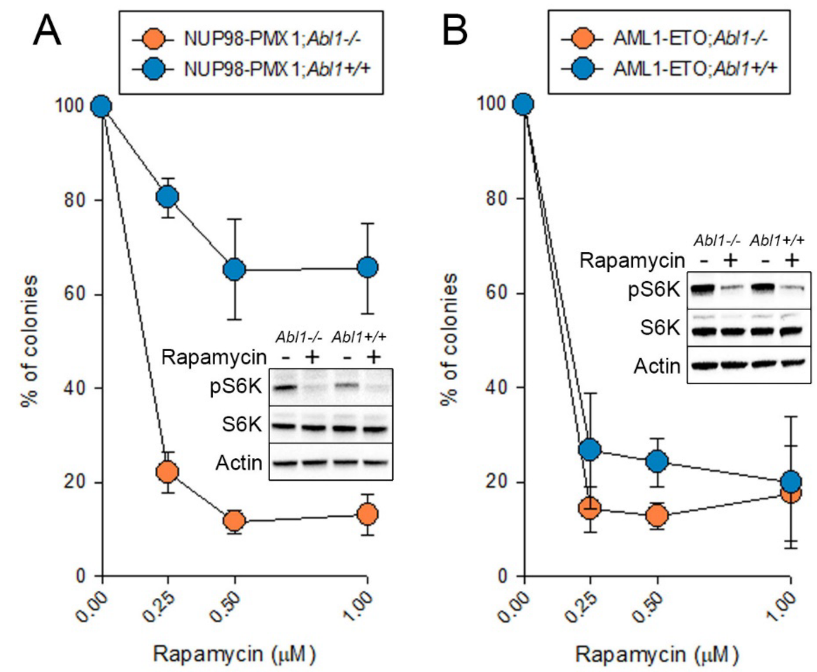

Supplemental Figure S3 (related to Figure 3). (**A**) Lin-c-Kit+ NUP98-PMX1;*Abl1-/-* and NUP98-PMX1;*Abl1+/+* cells, and (**B**) AML1-ETO;*Abl1-/-*, and AML1-ETO;*Abl1+/+* murine bone marrow cells were treated with the indicated concentrations of mTOR inhibitor rapamycin. After 72 hrs cells were plated in methylcellulose and colonies were counted 7 days later. Results represent mean % ± SD of colonies when compared to untreated controls. Insets: cells were treated with 0.25 μM rapamycin for 24 hrs. Total cell lysates were analyzed by Western blot detecting indicated proteins (p=phosphorylated).

Supplemental Table S1. Primers for mice genotyping.

**Target**	**Forward**	**Reverse**	**PCR product**
Abl1	CCTGGCCTCCAAGAGCAC	AGCCCCAGGGCATAGATAGT	*Abl1-/-* 210bp<br>*Abl1+/-* 210 bp and 162 bp<br>*Abl1+/+* 162 bp
Vav-Cre	GGTGTTGTAGTTGTCCCCACT	CAGGTTTTGGTGCACAGTCA	*iCre* 390 bp
Internal Positive Control (IPC)	CTAGGCCACAGAATTGAAAGATCT	GTAGGTGGAAATTCTAGCATCATCC	*IPC* 324 bp

Supplemental Table S2. gRNAs and primers specific for murine *Abl1*.

Target	cDNA	Forward	Reverse	PCR product(bp)
Mouse *Abl1*	gRNA1	CACCGCCCTTCATACCGCAGCGAGA	AAACTCTCGCTGCGGTATGAAGGGC	
	gRNA2	CACCGAAGGGAGGGTGTACCACTAC	AAACGTAGTGGTACACCCTCCCTTC	
	g1/2 PCR primer	ATGACGAGCTGCTTGGGTTG	CAGGACCAAGACCCCTTCTTC	455bp

Supplemental Table S3. KEGG Pathway analysis of the pathways deregulated in growth factor-independent 32Dcl3-*Abl1ko* cells.

Term	P value	Genes	Fold Enrichment	Bonferroni	Benjamini	FDR
mmu04151:PI3K-Akt signaling pathway	0.000226	Col6a3, Csf3, Epha2, Fgf13, Fgf18, Fgf6, Fgfr3, Gh, Il6ra, Itgb6, Thbs2, Tnc	3.810727115	0.02670194	0.026702	0.25857087
mmu01100:Metabolic pathways	0.011895	Aco1, Bst1, Cyp2j5, Cyp3a13, Fbp2, P4ha2, Pld2, Ptgs2, Hpgds, B4galt6, Plpp3, Bdh1, Aoc1, Gldc, B3gnt5, Dse, Chdh, Pip5kl1, A4galt, Plch1	1.756718174	0.76211431	0.512265	12.83487498
mmu05200:Pathways in cancer	0.022452	Ptgs2, Fgf13, Fgf18, Fgf6, Fgfr3, Agtr1a, Csf2ra, Ednra, Wnt4	2.539680793	0.93444774	0.596792	22.94628723
mmu04360:Axon guidance	0.026965	Epha2, Plxna1, Sema4f, Slit1, Plxnb1	4.32030109	0.96237919	0.55959	26.93254123
mmu04640:Hematopoietic cell lineage	0.039048	Csf2ra, Csf3, Il6ra, Cd14	5.245353794	0.99160114	0.615546	36.69634633
mmu04015:Rap1 signaling pathway	0.040317	Epha2, Fgf13, Fgf18, Fgf6, Fgfr3, Magi2	3.125152377	0.992833	0.560907	37.64958
mmu04512:ECM-receptor interaction	0.042587	Col6a3, Itgb6, Thbs2, Tnc	5.066534914	0.99460587	0.52577	39.32171626
mmu04014:Ras signaling pathway	0.05135	Epha2, Fgf13, Fgf18, Fgf6, Fgfr3, Pld2	2.920448073	0.99821057	0.546487	45.40008965

KEGG pathways enrichment analysis was performed using DAVID database. FDR refers to False discovery rate, p values were calculated using Fisher’s exact test, FDR was calculated using Benjamini and Hochberg in DAVID database.

Supplemental Table S4. Genes uniquely expressed in 32Dcl3-*Abl1ko*/IL-3- cells when compared to 32Dcl3/IL-3+ cells and 32Dcl3-*Abl1ko*/IL-3+ cells.

Gene name	Uniq_E1_E2_counts	C2 counts	Fold change E1-E2/C2	Log2Fold change
aconitase 1(Aco1)	2	6	0.333333333	-1.584962501
angiotensin II receptor, type 1a(Agtr1a)	8	2	4	2
ankyrin 3, epithelial(Ank3)	2	2	1	0
apolipoprotein A-I(Apoa1)	2	2	1	0
aquaporin 5(Aqp5)	2	2	1	0
bone gamma-carboxyglutamate protein 2(Bglap2)	10	14	0.714285714	-0.485426827
bone marrow stromal cell antigen 1(Bst1)	4	56	0.071428571	-3.807354922
B cell translocation gene 3(Btg3)	4	2	2	1
CD14 antigen(Cd14)	64	442	0.14479638	-2.787902559
cystic fibrosis transmembrane conductance regulator(Cftr)	4	14	0.285714286	-1.807354922
chitinase-like 3(Chil3)	2	4	0.5	-1
collagen, type VI, alpha 3(Col6a3)	8	8	1	0
crystallin, beta A4(Cryba4)	2	2	1	0
colony stimulating factor 2 receptor, alpha, low-affinity (granulocyte-macrophage)(Csf2ra)	2	8	0.25	-2
colony stimulating factor 3 (granulocyte)(Csf3)	2	6	0.333333333	-1.584962501
chemokine (C-X3-C motif) receptor 1(Cx3cr1)	2	2	1	0
cytochrome P450, family 2, subfamily j, polypeptide 5(Cyp2j5)	2	2	1	0
cytochrome P450, family 3, subfamily a, polypeptide 13(Cyp3a13)	2	2	1	0
cytochrome P450, family 4, subfamily b, polypeptide 1(Cyp4b1)	2	2	1	0
dipeptidylpeptidase 4(Dpp4)	2	2	1	0
endothelin receptor type A(Ednra)	2	2	1	0
erythrocyte membrane protein band 4.1 like 4a(Epb41l4a)	3	4	0.75	-0.415037499
Eph receptor A2(Epha2)	6	14	0.428571429	-1.222392421
fructose bisphosphatase 2(Fbp2)	2	6	0.333333333	-1.584962501
fibroblast growth factor 13(Fgf13)	2	4	0.5	-1
fibroblast growth factor 18(Fgf18)	6	4	1.5	0.584962501
fibroblast growth factor 6(Fgf6)	6	4	1.5	0.584962501
fibroblast growth factor receptor 3(Fgfr3)	2	2	1	0

neuronal calcium sensor 1(Ncs1)	2	2	1	0
growth hormone(Gh)	2	4	0.5	-1
heart and neural crest derivatives expressed transcript 2(Hand2)	6	2	3	1.584962501
interleukin 6 receptor, alpha(Il6ra)	2	8	0.25	-2
integrin beta 6(Itgb6)	2	4	0.5	-1
jagged 1(Jag1)	6	2	3	1.584962501
potassium voltage gated channel, Shab-related subfamily, member 1(Kcnb1)	2	2	1	0
potassium inwardly-rectifying channel, subfamily J, member 15(Kcnj15)	2	2	1	0
killer cell lectin-like receptor, subfamily A, member 2(Klra2)	18	90	0.2	-2.321928095
keratin 8(Krt8)	4	2	2	1
lysyl oxidase(Lox)	2	4	0.5	-1
leucine rich repeat protein 3, neuronal(Lrrn3)	2	4	0.5	-1
maternally expressed 3(Meg3)	2	4	0.5	-1
matrix metallopeptidase 8(Mmp8)	12	4	3	1.584962501
myocilin(Myoc)	2	6	0.333333333	-1.584962501
solute carrier family 11 (proton-coupled divalent metal ion transporters), member 1(Slc11a1)	10	52	0.192307692	-2.378511623
procollagen-proline, 2-oxoglutarate 4-dioxygenase (proline 4-hydroxylase), alpha II polypeptide(P4ha2)	10	6	1.666666667	0.736965594
proprotein convertase subtilisin/kexin type 5(Pcsk5)	2	2	1	0
plasminogen activator, tissue(Plat)	4	2	2	1
phospholipase D2(Pld2)	2	2	1	0
plexin A1(Plxna1)	2	2	1	0
prostaglandin-endoperoxide synthase 2(Ptgs2)	2	66	0.03030303	-5.044394119
S100 calcium binding protein A3(S100a3)	6	4	1.5	0.584962501
sema domain, immunoglobulin domain (Ig), TM domain, and short cytoplasmic domain(Sema4f)	2	10	0.2	-2.321928095
slit homolog 1 (Drosophila)(Slit1)	16	6	2.666666667	1.415037499
synuclein, gamma(Sncg)	2	2	1	0
SRY (sex determining region Y)-box 7(Sox7)	4	2	2	1
spectrin alpha, erythrocytic 1(Spta1)	28	48	0.583333333	-0.777607579
stefin A2(Stfa2)	190	524	0.36259542	-1.463567393

stimulated by retinoic acid gene 8(Stra8)	2	2	1	0
trefoil factor 2 (spasmolytic protein 1)(Tff2)	6	4	1.5	0.584962501
thrombospondin 2(Thbs2)	4	4	1	0
tenascin C(Tnc)	4	2	2	1
troponin T3, skeletal, fast(Tnnt3)	8	4	2	1
tropomyosin 2, beta(Tpm2)	2	6	0.333333333	-1.584962501
testis-specific serine kinase 1(Tssk1)	2	4	0.5	-1
dihydropyrimidinase-like 3(Dpysl3)	2	6	0.333333333	-1.584962501
wingless-type MMTV integration site family, member 4(Wnt4)	2	2	1	0
X-linked lymphocyte-regulated(Xlr)	6	10	0.6	-0.736965594
X-linked lymphocyte-regulated 3C(Xlr3c)	6	4	1.5	0.584962501
C-type lectin domain family 5, member a(Clec5a)	2	10	0.2	-2.321928095
urotensin 2(Uts2)	4	2	2	1
carcinoembryonic antigen-related cell adhesion molecule 9(Ceacam9)	4	2	2	1
C-type lectin domain family 4, member a2(Clec4a2)	4	16	0.25	-2
osteomodulin(Omd)	2	2	1	0
small nucleolar RNA, C/D box 34(Snord34)	4	2	2	1
HRAS-like suppressor(Hrasls)	2	2	1	0
ring finger protein 17(Rnf17)	2	2	1	0
membrane associated guanylate kinase, WW and PDZ domain containing 2(Magi2)	2	2	1	0
Iroquois related homeobox 4 (Drosophila)(Irx4)	2	2	1	0
BCL2-related ovarian killer(Bok)	4	6	0.666666667	-0.584962501
DNA segment, Chr 6, ERATO Doi 527, expressed(D6Ertd527e)	2	2	1	0
adhesion G protein-coupled receptor E4(Adgre4)	2	8	0.25	-2
hematopoietic prostaglandin D synthase(Hpgds)	8	30	0.266666667	-1.906890596
aldo-keto reductase family 1, member E1(Akr1e1)	2	2	1	0
dynein, axonemal, heavy chain 10(Dnah10)	6	4	1.5	0.584962501
DMRT-like family B with proline-rich C-terminal, 1(Dmrtb1)	2	6	0.333333333	-1.584962501

UDP-Gal:betaGlcNAc beta 1,4-galactosyltransferase, polypeptide 6(B4galt6)	2	6	0.333333333	-1.584962501
A kinase (PRKA) interacting protein 1(Akip1)	4	8	0.5	-1
cell cycle exit and neuronal differentiation 1(Cend1)	2	2	1	0
DnaJ heat shock protein family (Hsp40) member B7(Dnajb7)	2	2	1	0
MLX interacting protein-like(Mlxipl)	2	2	1	0
Ellis van Creveld gene syndrome(Evc)	2	2	1	0
kidney expressed gene 1(Keg1)	4	4	1	0
sushi, von Willebrand factor type A, EGF and pentraxin domain containing 1(Svep1)	2	2	1	0
transmembrane 4 L six family member 20(Tm4sf20)	2	2	1	0
RIKEN cDNA 4921522P10 gene(4921522P10Rik)	6	4	1.5	0.584962501
RIKEN cDNA 4933432I09 gene(4933432I09Rik)	4	6	0.666666667	-0.584962501
contactin associated protein-like 2(Cntnap2)	2	10	0.2	-2.321928095
RIKEN cDNA 1700109G14 gene(1700109G14Rik)	2	6	0.333333333	-1.584962501
EPS8-like 1(Eps8l1)	4	2	2	1
phospholipid phosphatase 3(Plpp3)	4	6	0.666666667	-0.584962501
RIKEN cDNA B230217C12 gene(B230217C12Rik)	2	2	1	0
RIKEN cDNA A930003A15 gene(A930003A15Rik)	30	64	0.46875	-1.093109404
RIKEN cDNA 4930565N06 gene(4930565N06Rik)	2	8	0.25	-2
myeloid-associated differentiation marker-like 2(Myadml2)	2	6	0.333333333	-1.584962501
cartilage intermediate layer protein 2(Cilp2)	4	10	0.4	-1.321928095
placenta expressed transcript 1, opposite strand(Plet1os)	2	2	1	0
muscular LMNA-interacting protein(Mlip)	4	4	1	0
membrane-spanning 4-domains, subfamily A, member 6B(Ms4a6b)	2	4	0.5	-1

RIKEN cDNA 1700028I16 gene(1700028I16Rik)	12	2	6	2.584962501
G protein-coupled receptor 137C(Gpr137c)	28	90	0.311111111	-1.684498174
family with sequence similarity 71, member D(Fam71d)	4	4	1	0
sperm flagellar 1(Spef1)	10	4	2.5	1.321928095
storkhead box 2(Stox2)	2	2	1	0
cyclin-dependent kinase-like 1 (CDC2-related kinase)(Cdkl1)	2	6	0.333333333	-1.584962501
Bardet-Biedl syndrome 7 (human)(Bbs7)	2	2	1	0
pogo transposable element with KRAB domain(Pogk)	6	2	3	1.584962501
armadillo repeat containing, X-linked 3(Armcx3)	2	2	1	0
LY6/PLAUR domain containing 6B(Lypd6b)	56	56	1	0
3-hydroxybutyrate dehydrogenase, type 1(Bdh1)	4	2	2	1
carcinoembryonic antigen-related cell adhesion molecule 18(Ceacam18)	2	8	0.25	-2
RIKEN cDNA 1700040L02 gene(1700040L02Rik)	4	2	2	1
RIKEN cDNA 1700110K17 gene(1700110K17Rik)	8	4	2	1
developmental pluripotency associated 2(Dppa2)	2	2	1	0
roundabout guidance receptor 4(Robo4)	4	2	2	1
zinc finger protein 946(Zfp946)	2	2	1	0
tubulin tyrosine ligase-like family, member 11(Ttll11)	2	4	0.5	-1
kelch repeat and BTB (POZ) domain containing 12(Kbtbd12)	2	2	1	0
RIKEN cDNA 4930512B01 gene(4930512B01Rik)	2	2	1	0
RAB3A interacting protein (rabin3)-like 1(Rab3il1)	6	2	3	1.584962501
MAM domain containing glycosylphosphatidylinositol anchor 1(Mdga1)	2	4	0.5	-1
RIKEN cDNA 4930503E14 gene(4930503E14Rik)	6	4	1.5	0.584962501
RIKEN cDNA 4930503B20 gene(4930503B20Rik)	2	6	0.333333333	-1.584962501

RIKEN cDNA 4930556J24 gene(4930556J24Rik)	6	6	1	0
RIKEN cDNA 4930598F16 gene(4930598F16Rik)	2	2	1	0
POU domain class 5, transcription factor 2(Pou5f2)	6	2	3	1.584962501
testis expressed 29(Tex29)	2	4	0.5	-1
spermatogenesis associated 45(Spata45)	8	2	4	2
coiled-coil domain containing 162(Ccdc162)	2	2	1	0
family with sequence similarity 131, member B(Fam131b)	2	4	0.5	-1
amine oxidase, copper-containing 1(Aoc1)	10	12	0.833333333	-0.263034406
metallophosphoesterase domain containing 2(Mpped2)	2	2	1	0
carbohydrate (N-acetylgalactosamine 4-sulfate 6-O) sulfotransferase 15(Chst15)	8	8	1	0
RIKEN cDNA 3300005D01 gene(3300005D01Rik)	4	6	0.666666667	-0.584962501
RIKEN cDNA C330022C24 gene(C330022C24Rik)	2	4	0.5	-1
nuclear receptor interacting protein 3(Nrip3)	6	2	3	1.584962501
hedgehog interacting protein-like 2(Hhipl2)	4	2	2	1
ankyrin repeat and SOCS box-containing 15(Asb15)	2	6	0.333333333	-1.584962501
ubiquitin-like domain containing CTD phosphatase 1(Ublcp1)	4	2	2	1
radial spoke head 6 homolog A (Chlamydomonas)(Rsph6a)	6	2	3	1.584962501
pellino 2(Peli2)	2	4	0.5	-1
expressed sequence C86187(C86187)	4	4	1	0
CKLF-like MARVEL transmembrane domain containing 4(Cmtm4)	2	2	1	0
transmembrane protein 132A(Tmem132a)	4	2	2	1
SLAM family member 9(Slamf9)	2	4	0.5	-1
expressed sequence AA414992(AA414992)	4	2	2	1
glycine decarboxylase(Gldc)	4	6	0.666666667	-0.584962501
lymphocyte antigen 6 complex pseudogene(9030619P08Rik)	20	12	1.666666667	0.736965594

leucine rich repeat transmembrane neuronal 2(Lrrtm2)	8	2	4	2
prolactin family 2, subfamily c, member 5(Prl2c5)	18	54	0.333333333	-1.584962501
glial cells missing homolog 2 (Drosophila)(Gcm2)	4	8	0.5	-1
UDP-GlcNAc:betaGal beta-1,3-N-acetylglucosaminyltransferase 5(B3gnt5)	2	10	0.2	-2.321928095
caspase recruitment domain family, member 11(Card11)	10	10	1	0
amyotrophic lateral sclerosis 2 (juvenile) chromosome region, candidate 12 (human)(Als2cr12)	2	8	0.25	-2
adenosine deaminase, RNA-specific, B1(Adarb1)	4	2	2	1
ankyrin repeat and SOCS box-containing 13(Asb13)	2	2	1	0
vomeronasal 1 receptor 21(Vmn1r21)	2	4	0.5	-1
BCL2/adenovirus E1B 19kD interacting protein like(Bnipl)	4	4	1	0
SKI family transcriptional corepressor 1(Skor1)	2	4	0.5	-1
pleckstrin homology like domain, family B, member 2(Phldb2)	4	20	0.2	-2.321928095
HUS1 checkpoint clamp component B(Hus1b)	2	4	0.5	-1
RIKEN cDNA 6720489N17 gene(6720489N17Rik)	2	2	1	0
potassium inwardly-rectifying channel, subfamily J, member 14(Kcnj14)	4	10	0.4	-1.321928095
ArfGAP with RhoGAP domain, ankyrin repeat and PH domain 2(Arap2)	2	2	1	0
dermatan sulfate epimerase(Dse)	2	2	1	0
WD repeat domain 49(Wdr49)	2	8	0.25	-2
cyclin O(Ccno)	4	6	0.666666667	-0.584962501
leucine rich repeat containing 3B(Lrrc3b)	6	2	3	1.584962501
choline dehydrogenase(Chdh)	12	34	0.352941176	-1.502500341
scavenger receptor class A, member 3(Scara3)	2	2	1	0
actin-binding Rho activating protein(Abra)	2	2	1	0
interferon activated gene 207(Ifi207)	4	2	2	1
calcium/calmodulin-dependent protein kinase ID(Camk1d)	2	2	1	0

phosphatidylinositol-4-phosphate 5-kinase-like 1(Pip5kl1)	28	6	4.666666667	2.222392421
LARGE xylosyl- and glucuronyltransferase 2(Large2)	2	2	1	0
adhesion molecule with Ig like domain 1(Amigo1)	4	2	2	1
tetratricopeptide repeat domain 22(Ttc22)	2	2	1	0
RIKEN cDNA 5730409E04Rik gene(5730409E04Rik)	2	2	1	0
grainyhead-like 3 (Drosophila)(Grhl3)	2	3	0.666666667	-0.584962501
transmembrane protein 159(Tmem159)	2	4	0.5	-1
plexin B1(Plxnb1)	6	2	3	1.584962501
cDNA sequence AF067061(AF067061)	4	24	0.166666667	-2.584962501
adhesion G protein-coupled receptor G2(Adgrg2)	6	10	0.6	-0.736965594
leucine rich repeat containing 3(Lrrc3)	6	2	3	1.584962501
alpha 1,4-galactosyltransferase(A4galt)	10	22	0.454545455	-1.137503524
Mab-21 domain containing 2(Mb21d2)	2	2	1	0
zinc finger protein 760(Zfp760)	2	4	0.5	-1
SET binding protein 1(Setbp1)	2	2	1	0
predicted gene 4955(Gm4955)	4	6	0.666666667	-0.584962501
ribonucleoprotein, PTB-binding 2(Raver2)	2	10	0.2	-2.321928095
hydroxysteroid (17-beta) dehydrogenase 13(Hsd17b13)	2	20	0.1	-3.321928095
olfactory receptor 1420(Olfr1420)	8	4	2	1
olfactory receptor 248(Olfr248)	12	2	6	2.584962501
olfactory receptor 414(Olfr414)	2	6	0.333333333	-1.584962501
phospholipase C, eta 1(Plch1)	4	2	2	1
C-type lectin domain family 4, member a1(Clec4a1)	2	8	0.25	-2
ADAMTS-like 3(Adamtsl3)	2	6	0.333333333	-1.584962501
Cd200 receptor 2(Cd200r2)	6	12	0.5	-1
ATP/GTP binding protein-like 2(Agbl2)	4	2	2	1
leucine rich repeat containing 4B(Lrrc4b)	8	2	4	2
transcription factor-like 5 (basic helix-loop-helix)(Tcfl5)	4	4	1	0
RIKEN cDNA E130310I04 gene(E130310I04Rik)	6	8	0.75	-0.415037499
tensin 3(Tns3)	2	4	0.5	-1
F-box and leucine-rich repeat protein 13(Fbxl13)	4	2	2	1

protein phosphatase 1E (PP2C domain containing)(Ppm1e)	6	8	0.75	-0.415037499
RIKEN cDNA 3222401L13 gene(3222401L13Rik)	4	4	1	0
transmembrane protein 117(Tmem117)	4	2	2	1
cytochrome P450, family 4, subfamily f, polypeptide 39(Cyp4f39)	12	10	1.2	0.263034406
heat shock transcription factor family member 5(Hsf5)	2	4	0.5	-1
RIKEN cDNA 9830107B12 gene(9830107B12Rik)	8	112	0.071428571	-3.807354922
transmembrane protein 52B(Tmem52b)	2	2	1	0
ring finger protein 150(Rnf150)	2	2	1	0
prostate and testis expressed 2(Pate2)	4	4	1	0
transmembrane protein 182(Tmem182)	4	2	2	1
predicted gene 13194(Gm13194)	6	1	6	2.584962501
tripartite motif-containing 55(Trim55)	4	6	0.666666667	-0.584962501
dynein regulatory complex subunit 1(Drc1)	2	2	1	0
protein arginine N-methyltransferase 8(Prmt8)	2	2	1	0
lemur tyrosine kinase 3(Lmtk3)	2	2	1	0
microRNA let7b(Mirlet7b)	8	6	1.333333333	0.415037499
sodium channel, type IV, beta(Scn4b)	4	2	2	1
zinc finger protein 652, opposite strand(Zfp652os)	4	12	0.333333333	-1.584962501
predicted gene, 17746(Gm17746)	2	4	0.5	-1
Riken cDNA D930007P13 gene(D930007P13Rik)	2	6	0.333333333	-1.584962501
tripartite motif-containing 30C(Trim30c)	2	2	1	0
IQ motif containing K(Iqck)	2	4	0.5	-1
pluripotency associated transcript 25(Platr25)	2	2	1	0
RIKEN cDNA A230083G16 gene(A230083G16Rik)	2	2	1	0
glutamate rich 6(Erich6)	4	4	1	0
ankyrin repeat domain 34A(Ankrd34a)	2	6	0.333333333	-1.584962501
predicted gene 13141(Gm13141)	2	2	1	0
RIKEN cDNA 2610042L04 gene(2610042L04Rik)	2	2	1	0
RIKEN cDNA 2900079G21 gene(2900079G21Rik)	8	4	2	1
predicted gene 15133(Gm15133)	2	2	1	0

predicted gene 6408(Gm6408)	2	2	1	0
predicted gene 11992(Gm11992)	2	2	1	0
predicted pseudogene 6897(Gm6897)	4	4	1	0
predicted gene 12250(Gm12250)	2	2	1	0
C-type lectin domain family 2, member L(Clec2l)	16	2	8	3
predicted gene 8273(Gm8273)	8	10	0.8	-0.321928095
zinc finger protein pseudogene(Gm10416)	2	2	1	0
histocompatibility 60b(H60b)	4	4	1	0
predicted gene 11789(Gm11789)	2	4	0.5	-1
predicted gene 8817(Gm8817)	4	2	2	1
kinesin family member 26A(Kif26a)	2	2	1	0
predicted gene 9525(Gm9525)	6	18	0.333333333	-1.584962501
microRNA 93(Mir93)	4	4	1	0
microRNA 450b(Mir450b)	4	8	0.5	-1
microRNA 761(Mir761)	4	12	0.333333333	-1.584962501
predicted gene 10248(Gm10248)	6	2	3	1.584962501
zinc finger protein 389(Zfp389)	2	2	1	0
DNA methyltransferase 3A, opposite strand(Dnmt3aos)	2	2	1	0
predicted gene 10822(Gm10822)	12	16	0.75	-0.415037499
predicted gene 16503(Gm16503)	4	4	1	0
predicted gene 10864(Gm10864)	4	2	2	1
butyrophilin-like 1(Btnl1)	2	2	1	0
predicted gene 2666(Gm2666)	8	4	2	1
tandem duplication of RIKEN cDNA 1700049E17 gene, gene 2(1700049E17Rik2)	4	2	2	1
predicted gene 3916(Gm3916)	4	6	0.666666667	-0.584962501
vomeronasal 2, receptor 46(Vmn2r46)	4	12	0.333333333	-1.584962501
microRNA 343(Mir343)	2	2	1	0
RIKEN cDNA A230020J21 gene(A230020J21Rik)	2	2	1	0
small nucleolar RNA, C/D box 68(Snord68)	10	12	0.833333333	-0.263034406
predicted gene 12238(Gm12238)	4	2	2	1
RIKEN cDNA 9530027J09 gene(9530027J09Rik)	18	2	9	3.169925001
predicted gene, 19279(Gm19279)	2	6	0.333333333	-1.584962501
predicted gene 13752(Gm13752)	2	6	0.333333333	-1.584962501
predicted gene, 19658(Gm19658)	2	2	1	0
predicted gene, 19665(Gm19665)	8	4	2	1

predicted gene, 19673(Gm19673)	2	4	0.5	-1
predicted gene, 19691(Gm19691)	2	10	0.2	-2.321928095
predicted gene 11827(Gm11827)	6	14	0.428571429	-1.222392421
predicted gene 20603(Gm20603)	2	2	1	0
predicted gene, 19816(Gm19816)	10	4	2.5	1.321928095
uncharacterized protein C2orf16-like(LOC100503696)	4	10	0.4	-1.321928095
predicted gene 15558(Gm15558)	2	2	1	0
predicted gene, 19967(Gm19967)	10	2	5	2.321928095
predicted gene 17227(Gm17227)	2	4	0.5	-1
expressed sequence AA666905(AA666905)	2	2	1	0
predicted gene 15798(Gm15798)	4	2	2	1
predicted gene, 20385(Gm20385)	2	1	2	1
predicted gene, 25018(Gm25018)	2	2	1	0
